# Prostate Cancer Radiotherapy: Increased Biochemical Control and Late Toxicity in Men With Medication Allergies

**DOI:** 10.1093/jncics/pkaa081

**Published:** 2020-09-11

**Authors:** William Tyler Turchan, Stanley I Gutiontov, Michael T Spiotto, Stanley L Liauw

**Affiliations:** Department of Radiation and Cellular Oncology, University of Chicago, Chicago, IL, USA; Department of Radiation and Cellular Oncology, University of Chicago, Chicago, IL, USA; Department of Radiation Oncology, University of Texas M.D. Anderson Cancer Center, Houston, TX, USA; Department of Radiation and Cellular Oncology, University of Chicago, Chicago, IL, USA

## Abstract

**Background:**

Given similarities in the mediators of medication allergy (MA) and tissue response to radiotherapy, we assessed whether outcomes following prostate radiotherapy differ in patients with MAs.

**Methods:**

A total 587 men with known MA history and nonmetastatic prostate cancer underwent radiotherapy from 1989 to 2006. Clinicopathologic and treatment variables were analyzed for association with freedom from biochemical failure (FFBF) and late treatment–related, physician-defined Radiation Therapy Oncology Group gastrointestinal (GI) and genitourinary (GU) toxicity. Covariates identified on univariate analysis for toxicity and disease control were examined on multivariable analysis. All statistical tests were 2-sided, and a *P* less than .05 was considered statistically significant.

**Results:**

A total of 155 of 587 men (26.4%) had 1 or more MAs, most commonly to penicillin (n = 71), sulfa (n = 35), and aspirin or nonsteroidal antiinflammatory drugs (n = 28). On univariate analysis, men with MAs had superior 10-y FFBF (71.5% vs 63.5%, *P =* .02) and higher incidence of late GI grade 2 or higher (G2+; 20.6% vs 13.2%, *P =* .04) and grade 3 or higher (G3+; 7.5% vs 3.9%, *P =* .08) as well as late GU G2+ (42.5% vs 33.2%, *P =* .04) and G3+ (7.5% vs 3.0%, *P =* .02) toxicity than men without MAs. On multivariable analysis, MA history remained a statistically significant predictor of FFBF (hazard ratio [HR] = 0.64, 95% confidence interval [CI] = 0.43 to 0.93, *P =* .02), late G2+ GI (HR = 1.76, 95% CI = 1.06 to 2.90, *P=*.03), and G3+ GU (HR = 2.69, 95% CI = 1.16 to 6.27, *P =* .02) toxicity after controlling for corresponding covariates in each model.

**Conclusions:**

Men with MAs had improved FFBF and increased treatment-related toxicity following radiotherapy for prostate cancer. MA history could be a relevant consideration in the management of men with localized prostate cancer.

With more than 190 000 new diagnoses each year, prostate cancer is the most commonly diagnosed noncutaneous malignancy in American men (1). The vast majority of men diagnosed with prostate cancer have localized disease. Local therapy is the mainstay of treatment in patients with nonmetastatic prostate cancer who either refuse active surveillance or are not good candidates for active surveillance based on the aggressiveness of their disease relative to their comorbidities and life expectancy. Standard-of-care local therapy options for intact prostate cancer include surgery and radiation therapy (RT), which can be delivered in the form of external beam radiation therapy (EBRT) or brachytherapy (BT) (2).

Given the relative dearth of high-quality evidence supporting either option as superior with respect to oncologic outcomes, surgery and RT are both generally accepted as appropriate local therapy options for patients with localized prostate cancer (2). As a result, potential toxicities and functional outcomes are important considerations for patients when deciding among therapeutic approaches. Genitourinary (GU) and gastrointestinal (GI) side effects are among the most common complications experienced by men receiving primary RT for prostate cancer. Late-GU toxicity typically manifests as increased urinary frequency, urgency, dysuria, and/or hematuria, and late GI toxicity typically manifests as increased bowel frequency, urgency, and/or blood in the stool. The Radiation Therapy Oncology Group has published criteria by which physicians grade acute and late treatment–related toxicity to quantify these outcomes (3), with reported rates of late GI or GU grade 2 or higher (G2+; symptoms not affecting lifestyle, responding to simple outpatient management) and grade 3 or higher (G3+; symptoms affecting lifestyle and often requiring minor procedures or hospital admission) on the order of 10%-30% and 2%-5%, respectively (4–7). Although these estimates approximate the risk of late treatment–related toxicity in the population as a whole, an individual patient’s risk of late toxicity is influenced by a number of factors specific to the patient and the RT delivered.

Several patient-specific comorbid factors have been linked to late treatment–related toxicity in patients receiving RT for prostate cancer. For example, increased rates of late GU toxicity have been demonstrated among patients with diabetes mellitus (DM) (8) and those who have undergone previous transurethral resection of the prostate (9), and late GI toxicity has been linked to patient age (10) and systemic anticoagulation (AC) (11). Although these and other comorbid factors have been demonstrated to influence rates of late treatment–related toxicity in men undergoing primary RT for prostate cancer, the impact of medication allergy (MA) has not been explored. MA occurs in approximately 3%-5% of hospitalized patients; however, the true incidence of MAs in the ambulatory population remains unknown (12). Although the mechanisms underlying MAs are complex and not entirely understood, given similarities in the hypothesized mediators of MAs (13) and late treatment–related toxicity (14), it is plausible that patients with MAs may be more likely to develop late treatment–related toxicity. As a result, we evaluated whether, among a cohort of prostate cancer patients treated with RT, patients with MAs have a higher rate of late treatment–related toxicity compared with patients without MAs. Moreover, given the demonstrated role of the immune response in MAs (13) and radiation-mediated tumor control (15), we hypothesized that patients with MAs may have differences in long-term disease control following primary RT compared with patients without MAs.

## Methods

### Determination of Clinicopathologic and Treatment Characteristics

A prospectively maintained database was used to retrospectively identify 587 men with nonmetastatic prostate cancer treated with primary RT at our institution from 1989 to 2006. Informed consent for inclusion in the database was obtained from all patients receiving ongoing follow-up. This study was approved by the institutional review board of the Chicago University Medical Center. MA history was documented and available for all men in the medical chart at initial consultation, reported by the patient, and verified in the chart across other providers. In the event that patient-reported symptoms were more consistent with medication intolerance or a side effect related to a medication rather than a true allergy, the reaction was not treated as a MA for the purposes of this study. Additional clinicopathologic and treatment variables commonly associated with treatment-related toxicity and disease control were recorded in the medical chart and the prospectively maintained patient database.

### Assessment of Outcomes

Men underwent routine clinical assessment at 6 weeks following the completion of RT followed by every 6-9 months for the first 5 years after the completion of RT with subsequent assessments performed annually. Toxicity was evaluated and recorded at the time of each assessment. Late toxicity was defined as toxicity occurring more than 3 months following the completion of RT. Toxicity was graded using a modified Radiation Therapy Oncology Group grading system, as was previously described: grade 1, minor GU or GI symptoms not requiring medical therapy; grade 2, moderate GU or GI symptoms requiring medication; grade 3, severe GU or GI symptoms requiring a procedure or intervention; grade 4, potentially life-threating GU or GI symptoms (8). Biochemical failure (BF) was defined using the Phoenix criteria (16).

### Statistical Analysis

Comparisons of clinicopathologic or treatment factors by MA history were performed using Pearson’s χ^2^ and Wilcoxon tests. The cumulative incidence of late GU and GI G2+ and G3+ toxicity was determined. Logistic regression was performed, and likelihood ratios were used to test for association between clinicopathologic or treatment factors and late toxicity. Covariates associated (*P* < .15) with late toxicity on univariate analysis (UVA) were subsequently included on multivariable analysis (MVA). Freedom from BF (FFBF), freedom from distant metastasis (FFDM), and prostate cancer-specific survival (PCSS) were determined by the Kaplan-Meier method with log-rank tests performed for comparisons on UVA; the Cox method was used on MVA, and the assumption of proportional hazards was confirmed graphically. All statistical tests were 2-sided, and a *P* less than .05 was considered statistically significant. All statistical analysis was performed using JMP, Version 14 (SAS Institute Inc, Cary, NC).

## Results

### Patient and Treatment Characteristics

The median age was 68 years. The median prostate volume was 41 cm^3^. A total of 105 men (17.9%) had a diagnosis of DM and 43 men (7.3%) were on AC. The National Comprehensive Cancer Network (NCCN) risk category was low, intermediate, and high in 209 (35.7%), 225 (38.5%), and 151 (25.8%) men, respectively. Patient characteristics are further detailed in Table 1.

**Table 1.** Patient and treatment characteristics

Patient and treatment characteristics	All men (n = 587)	MA (n = 155)	No MA (n = 432)	*P* ^a^
Median pretreatment PSA, ng/mL (IQR)	8 (6-16)	8 (5-15)	9 (6-16)	.02
Clinical T-stage T1a-T1c T2a-T2c T3a-T3b	473 (80.9) 79 (13.5) 33 (5.6)	127 (82.5) 21 (13.6) 6 (3.9)	346 (80.3) 58 (13.5) 27 (6.3)	.55
Clinically node-positive, No. (%)	5 (0.9)	0 (0.0)	5 (1.1)	.03
ISUP grade group, No. (%) 1 2 3 4 5	351 (64.8) 99 (18.3) 42 (7.8) 36 (6.7) 13 (2.4)	92 (62.2) 29 (19.6) 16 (10.8) 9 (6.1) 2 (1.4)	259 (65.9) 70 (17.8) 26 (6.7) 27 (6.9) 11 (2.8)	.43
NCCN risk category, No. (%) Low Intermediate High	209 (35.7) 225 (38.4) 151 (25.8)	62 (40.0) 56 (36.2) 37 (23.8)	147 (34.2) 169 (39.3) 114 (26.5)	.43
Treatment modality, No. (%) EBRT alone Brachy alone EBRT + Brachy^b^	511 (87.1) 44 (7.5) 32 (5.5)	130 (83.9) 13 (8.4) 12 (7.7)	381 (88.2) 31 (7.2) 20 (4.6)	.29
Median EBRT dose, Gy (IQR)	72.6 (70-76)	74 (70-76)	72 (70-76)	.18
IMRT, No. (%)	255 (46.9)	73 (51.4)	182 (45.3)	.29
ADT, No. (%)	249 (42.4)	66 (42.6)	183 (42.4)	.96
Median ADT duration, mo (IQR)	4 (3-4)	4 (4-4)	4 (3-4)	.25
Median age, y (IQR)	68 (63-73)	69 (64-74)	68 (63-72)	.046
Anticoagulation, No. (%)	43 (7.3)	12 (7.8)	31 (7.2)	.82
Diabetes mellitus, No. (%)	105 (17.9)	40 (26.0)	65 (15.0)	.002
Median prostate volume, cm^3^ (IQR)	41 (31-53)	42 (32-50)	41 (30-56)	.64
Median follow-up length, mo (IQR)	113 (60-153)	126 (74-156)	108 (55-152)	.047

a Comparisons of patient or treatment characteristics by MA history were performed using Pearson’s χ^2^ and Wilcoxon tests. All statistical tests were 2-sided, and a *P* less than .05 was considered statistically significant. ADT = androgen deprivation therapy; EBRT = external beam radiation therapy; IMRT = intensity-modulated radiation therapy; IQR = interquartile range; ISUP = International Society of Urological Pathology; MA = medication allergy; NCCN = National Comprehensive Cancer Network; PSA = prostate-specific antigen.

b brachytherapy.

Treatment modality included EBRT (median dose=72.6 Gy), BT (median dose=144 Gy), and EBRT + BT (median dose=153 Gy) in 511 (87.1%), 44 (7.5%), and 32 (5.5%) men, respectively. A total of 324 men (55.2%) were treated with dose-escalated RT (EBRT dose ≥74 Gy or BT). Intensity-modulated radiation therapy (RT) was used to treat 255 (46.9%) of the men treated with EBRT or EBRT + BT. Thirty-six (6.7%) of the men treated with EBRT or EBRT + BT received whole pelvic RT. Also, 249 (42.4%) men received concurrent androgen deprivation therapy (ADT; median time 4 months). Additional details regarding treatment patterns are shown in Table 1.

A total of 155 men (26.4%) had 1 or more MAs with a median of 1 MA (interquartile range = 1-2) per individual. Forty-seven (8.0%) men had 2 or more MAs. The most common recorded MAs were penicillin (n = 71), sulfa (n = 35), and aspirin or nonsteroidal anti-inflammatory drugs (n = 28). Men with MAs were slightly older (median age 69 years vs 68 years, *P =* .046) and more likely to have DM (26.0% vs 15.1%, *P =* .002) compared with men with no MAs, but were similar with regard to prostate volume and frequency of AC (Table 1). Men with MAs had marginally lower pretreatment prostate-specific antigen (PSA) levels (median 8 ng/mL vs 9 ng/mL, *P* = .02) and were less likely to be clinically node-positive (0.0% vs 1.2%, *P* = .03), but otherwise did not differ from men with MAs with respect to disease or treatment characteristics (Table 1). The median follow-up was slightly longer in men with MAs compared with those without MAs (median 126 months vs 108 months, *P =* .047).

### Late Treatment–Related Toxicity

With a median follow-up of 113 months, the cumulative incidence of late GU G2+ and G3+ toxicity was 35.6% and 4.2%, respectively. Cumulative incidence of late GI G2+ and G3+ toxicity was 15.2% and 4.9%, respectively. On UVA, MA was associated with increased risk of late GU G2+ toxicity (42.5% vs 33.2%, *P =* .04) and G3+ toxicity (7.5% vs 3.0%, *P =* .02) as well as late GI G2+ toxicity (20.6% vs 13.2%, *P =* .04) and G3+ toxicity (7.5% vs 3.9%, *P =* .08), as shown in Table 2. Covariates associated with late GU G2+ toxicity on UVA included treatment modality, RT dose equal to or greater than 74 Gy, and intensity-modulated RT; only treatment modality was associated with late GU G3+ toxicity (Table 2). Covariates associated with late GI G2+ toxicity on UVA included AC, ADT, and age 70 years or older; only ADT was associated with late GI G3+ toxicity (Table 2). Results of MVAs for late toxicity are shown in Table 3. On MVA, history of MA (hazard ratio [HR] = 2.69, 95% confidence interval [CI] = 1.16 to 6.27, *P =* .02) was associated with late GU G3+ toxicity after accounting for BT boost (EBRT vs EBRT + BT; HR = 1.60, 95% CI = 0.35 to 7.31, *P =* .55). MA was also associated with late GI G2+ toxicity (HR = 1.76, 95% CI = 1.06 to 2.90, *P =* .03) after accounting for ADT (HR = 0.61, 95% CI = 0.37 to 1.01, *P =* .05), age 70 years and older (HR = 1.45, 95% CI = 0.91 to 2.34, *P =* .12), and AC (HR = 3.21, 95% CI = 1.56 to 6.60, *P =* .002).

**Table 2.** Univariate analyses for late GU and GI toxicity

Patient and treatment characteristics	Late GU G2+		Late GU G3+		Late GI G2+		Late GI G3+	
	Cumulative incidence, %	*P* ^a^	Cumulative incidence, %	*P* ^a^	Cumulative incidence, %	*P* ^a^	Cumulative incidence, %	*P* ^a^
Treatment modality EBRT alone vs Brachy alone EBRT alone vs EBRT + Brachy^b^ Brachy alone vs EBRT + Brachy^b^	34.0 vs 25.0 34.0 vs 77.8 25.0 vs 77.8	.30 <.001 <.001	4.3 vs 0.0 4.3 vs 7.4 0.0 vs 7.4	.23 .44 .12	15.6 vs 9.4 15.6 vs 14.8 9.4 vs 14.8	.35 .92 .52	4.7 vs 6.3 4.7 vs 7.4 6.3 vs 7.4	.68 .51 .86
EBRT dose								
<74 vs ≥74 Gy	29.3 vs 39.1	.02	3.6 vs 4.9	.47	16.0 vs 15.2	.81	5.6 vs 3.7	.32
IMRT								
Yes vs no	40.4 vs 31.7	.03	4.8 vs 3.6	.49	14.8 vs 15.5	.83	3.6 vs 5.9	.21
ADT								
Yes vs no	38.7 vs 33.3	.20	5.0 vs 3.5	.37	11.8 vs 17.7	.05	2.9 vs 6.3	.07
Age, y								
<70 vs ≥70	36.0 vs 35.2	.84	4.1 vs 4.2	.98	13.0 vs 18.0	.11	5.9 vs 4.1	.35
Anticoagulation								
Yes vs no	30.8 vs 36.0	.51	2.6 vs 4.3	.61	33.3 vs 13.8	.001	7.7 vs 4.7	.40
Diabetes mellitus								
Yes vs no	41.6 vs 34.4	.17	5.9 vs 3.8	.32	14.9 vs 15.3	.92	4.0 vs 5.1	.63
Prostate volume								
<40 vs ≥40 cm^3^	41.0 vs 37.0	.45	5.0 vs 4.1	.68	12.4 vs 17.9	.16	5.6 vs 3.5	.35
MA								
Yes vs no	42.5 vs 33.2	.04	7.5 vs 3.0	.02	20.6 vs 13.2	.04	7.5 vs 3.9	.08

a Logistic regression was performed, and likelihood ratios were used to test for association between clinicopathologic or treatment factors and late toxicity. All statistical tests were 2-sided, and a *P* less than .05 was considered statistically significant. ADT = androgen deprivation therapy; EBRT = external beam radiation therapy; G2 or G3 = grade 2 or higher toxicity or grade 3 or higher toxicity; GI = gastrointestinal; GU = genitourinary; IMRT = intensity-modulated radiation therapy; MA = medication allergy.

b brachytherapy

**Table 3.** Multivariable analyses for late GU and GI toxicity

Patient and treatment characteristics	RR (95% CI)	*P* ^a^
Late GU grade 2+ toxicity Treatment modality EBRT alone vs Brachy alone EBRT alone vs EBRT + brachy Brachy alone vs EBRT + brachy EBRT dose ≥74 Gy IMRT MA	0.59 (0.20 to 1.75) 5.90 (2.05 to 16.97) 9.95 (2.90 to 34.04) 1.33 (0.64 to 2.77) 1.18 (0.58 to 2.42) 1.44 (0.96 to 2.15)	.35 .001 <.001 .44 .65 .07
Late GU grade 3+ toxicity Treatment modality EBRT alone vs Brachy alone EBRT alone vs EBRT + brachy Brachy alone vs EBRT + brachy MA	—^b^ 1.60 (0.35 to 7.31) —^b^ 2.69 (1.16 to 6.27)	.98 .55 .99 .02
Late GI grade 2+ toxicity ADT Age ≥70 y Anticoagulation MA	0.61 (0.37 to 1.01) 1.45 (0.91 to 2.34) 3.21 (1.56 to 6.60) 1.76 (1.06 to 2.90)	.05 .12 .002 .03
Late GI grade 3+ toxicity ADT MA	2.26 (0.98 to 5.84) 2.02 (0.89 to 4.45)	.06 .09

a Logistic regression was performed, and likelihood ratios were used to test for association between clinicopathologic or treatment factors, identified on respective univariate analyses, and late toxicity in multivariable models. All statistical tests were 2-sided and a *P* less than .05 was considered statistically significant. ADT = androgen deprivation therapy; Brachy = brachytherapy; CI = confidence interval; EBRT = external beam radiation therapy; GI = gastrointestinal; GU = genitourinary; IMRT = intensity-modulated radiation therapy; MA = medication allergy; RR = risk ratio.

b No late GU grade 3+ events occurred in men treated with Brachy alone.

### Prostate-Cancer Specific Outcomes

A total of 166 men experienced BF with a median time to BF of 48 months. Ten-year FFBF was 65.7%. Forty-five men developed distant metastasis with a median time to distant metastasis of 36 months. Ten-year FFDM was 91.0%. Twenty-seven men experienced prostate cancer mortality with a median time to prostate cancer mortality of 67 months. Ten-year PCSS was 94.7%.

**Figure 1.**
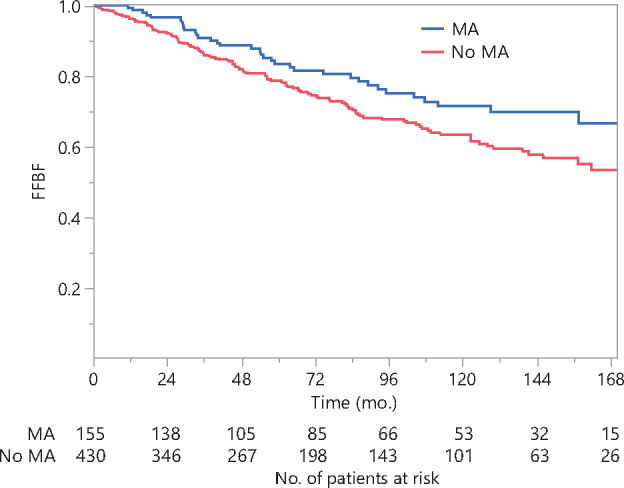
Freedom  from biochemical failure (FFBF) following primary radiation therapy stratified by medication allergy (MA) history. FFBF was determined by the Kaplan-Meier method with a 2-sided log-rank test performed to compare between groups. Men with 1 or more MAs had a 10-year FFBF of 71.5%, whereas men without MAs had a 10-year FFBF of 63.5% (*P* = .02).

FFBF was associated with multiple clinicopathologic factors on UVA (Table 4), including: pretreatment PSA, clinical T-stage, clinical N-stage, International Society of Urological Pathology grade group, NCCN risk category, treatment modality, dose escalation, and MA. By the Kaplan-Meier method, 10-year FFBF was 71.5% in men with MAs and 63.5% in men without MAs (Figure 1; *P =* .02). The improvement in FFBF among men with MAs was more prominent in men treated with ADT (*P* = .03) compared with men treated without ADT (*P* = .26). There was no statistically significant difference in FFBF among men with multiple MAs compared with men with 1 MA (*P* = .42).

**Table 4.** Univariate and multivariable analyses for 10-year FFBF (n = 587)

Patient and treatment characteristics	Univariate analysis		Multivariable analysis			
			Model 1		Model 2	
	10-y FFBF, %	*P* ^a^	HR (95% CI)	*P* ^a^	HR (95% CI)	*P* ^a^
Pretreatment PSA <10 vs 10 to <20 ng/mL <10 vs ≥20 ng/mL 10 to <20 vs ≥20 ng/mL	75.5 vs 61.0 75.5 vs 40.6 61.0 vs 40.6	.03 <.001 <.001	— — —	— — —	0.82 (0.53 to 1.29) 0.37 (0.24 to 0.57) 0.46 (0.28 to 0.74)	.38 <.001 .001
Clinical T-stage T1a-T1c vs T2a-T2c T1a-T1c vs T3a-T3b T2a-T2c vs T3a-T3b	71.7 vs 43.2 71.7 vs 36.1 43.2 vs 36.1	<.001 <.001 .58	— — —	— — —	0.66 (0.43 to 1.04) 0.40 (0.23 to 0.75) 0.61 (0.33 to 1.21)	.08 .006 .15
Clinically node-positive No vs yes	65.1 vs 50.0	.15	0.78 (0.19 to 2.14)	.67	0.65 (0.14 to 2.09)	.50
ISUP grade group 1 vs 2 1 vs 3 1 vs 4 1 vs 5 2 vs 3 2 vs 4 2 vs 5 3 vs 4 3 vs 5 4 vs 5	69.7 vs 62.9 69.7 vs 61.7 69.7 vs 50.2 69.7 vs 61.4 62.9 vs 61.7 62.9 vs 50.2 62.9 vs 61.4 61.7 vs 50.2 61.7 vs 61.4 50.2 vs 61.4	.39 .32 .005 .06 .86 .07 .20 .19 .33 .88	— — — — — — — — — —	— — — — — — — — — —	0.85 (0.55 to 1.35) 0.63 (0.35 to 1.15) 0.73 (0.41 to 1.31) 0.60 (0.23 to 1.58) 0.74 (0.37 to 1.45) 0.85 (0.44 to 1.63) 0.70 (0.26 to 1.89) 1.15 (0.54 to 2.48) 0.95 (0.33 to 2.75) 0.82 (0.28 to 2.41)	.51 .15 .31 .32 .39 .63 .49 .72 .92 .72
NCCN risk category Low vs intermediate Low vs high Intermediate vs high	82.1 vs 65.9 82.1 vs 43.1 65.9 vs 43.1	.004 <.001 <.001	0.55 (0.35 to 0.84) 0.25 (0.16 to 0.38) 0.45 (0.31 to 0.65)	.005 <.001 <.001	— — —	— — —
Treatment modality EBRT alone vs Brachy alone EBRT alone vs EBRT + Brachy Brachy alone vs EBRT + Brachy	64.7 vs 74.6 64.7 vs 69.2 74.6 vs 69.2	.43 .27 .89	— — —	— — —	— — —	— — —
Dose escalation (EBRT dose ≥74 Gy/Brachy) Yes vs no	76.3 vs 53.8	<.001	0.60 (0.43 to 0.83)	.002	0.56 (0.39 to 0.81)	.002
ADT Yes vs no	65.1 vs 66.2	.20	—	—	—	—
MA Yes vs no	71.5 vs 63.5	.02	0.64 (0.43 to 0.93)	.019	0.60 (0.39 to 0.88)	.009

a FFBF was determined by the Kaplan-Meier method with log-rank tests performed for comparisons on univariate analyses; the Cox method was used for multivariable analyses. All statistical tests were 2-sided, and a *P* less than .05 was considered statistically significant. ADT = androgen deprivation therapy; Brachy = brachytherapy; CI = confidence interval; EBRT = external beam radiation therapy; FFBF = freedom from biochemical failure; HR = hazard ratio; ISUP = International Society of Urological Pathology; MA = medication allergy; NCCN = National Comprehensive Cancer Network; PSA = prostate-specific antigen.

On MVA, MA remained associated with FFBF when controlling for clinical N-stage and dose escalation as well as both NCCN risk category (HR = 0.64, 95% CI = 0.43 to 0.93, *P* = .02; Table 4, Model 1) and pretreatment PSA, clinical T-stage, and International Society of Urological Pathology grade group (HR = 0.60, 95% CI = 0.39 to 0.88, *P* = .009; Table 4, Model 2). FFDM was associated with multiple factors on UVA (Supplementary Table 1, available online). Ten-year FFDM was 95.4% for men with MAs and 89.3% for men without MAs (*P* = .10). Accounting for other covariates, MA was not associated with FFDM (Supplementary Table 1, available online) or PCSS (Supplementary Table 2, available online) on MVA.

## Discussion

This study suggests that men with MAs may experience different long-term outcomes following primary RT for prostate cancer, including a twofold higher risk of late G2-3+ treatment–related morbidity as well as a twofold higher probability of long-term disease control. Differences in outcomes following RT may be related to intersecting mechanisms underlying MAs and tissue response to RT.

MAs are thought to be the result of a variety of medication and patient-specific factors. Among other mechanisms, T-cell–mediated responses are thought to play an important role in hypersensitivity reactions in patients with MAs (17). To mount a T-cell response, costimulatory signals beyond T-cell receptor stimulation are required. It has been hypothesized that MAs are, at least partially, the result of excessive expression of costimulatory signals, which lower the threshold for a T-cell response to occur (13); similar hypotheses have been formulated to explain the excess of potentially transient allergic responses to medications in patients with HIV and other viral infections (18). T-cell–mediated responses have similarly been implicated in the response to ionizing radiation, with decreased therapeutic response in immune-excluded tumors that lack T-cell infiltration (15,19,20). Given that patients with MAs may have a decreased threshold to mount a T-cell response, the improved FFBF observed among patients with MAs could be explained by increased antitumor immune surveillance. This phenomenon has previously been implicated as a potential explanation for decreased risk of lymph node metastasis in rectal cancer patients with MAs (21) and improved cancer mortality in patients with asthma and hay fever (22). This decreased threshold to mount a T-cell–mediated response may also explain the observed difference in late toxicity following RT among patients with MAs. Enhanced immune response may augment RT-mediated stromal stem cell depletion in patients with MAs, resulting in higher rates of late treatment–related toxicity (14). Additionally, the adaptive immune response triggers recruitment of a variety of immune cell populations via pro-inflammatory signals and the expression of several cytokines, including transforming growth factor beta (TGF-β). TGF-β expression is widely considered to be an important mediator of post-RT fibrosis (23–33) and has been implicated in radiation proctitis (34,35) and cystitis (29,36). In patients with scleroderma, increased baseline TGF-β expression has been implicated in the increased late treatment–related toxicity observed when these patients are treated with RT (37–39). Analogously, the decreased threshold to mount a T-cell–mediated response and the resulting increase in TGF-β expression in patients with MAs may explain the increase in late treatment–related toxicity observed among men with MAs in our cohort.

Although there is compelling evidence to support differences in the adaptive immune response among patients with MAs as the mechanism driving our observed outcomes, other factors may also explain this relationship. Eosinophils may also play an important role in late RT–related toxicity (40) and have been implicated in severe drug hypersensitivity (41). Likewise, mast cells, which have been suggested to be radioresistant (42) and play a role in late RT–related toxicity (43,44), are a known mediator of drug-induced anaphylaxis (45). Thus, it is conceivable that effectors of the innate immune response may also contribute to the increased RT-related toxicity observed in patients with MAs.

Nonimmune-mediated mechanisms may also play a role in MAs and contribute to the increase in late RT–related toxicity in this population. For example, RhoA-ROCK signaling, which has been demonstrated to increase in normal tissues following prostate RT, can be targeted with inhibitors to decrease late RT–related toxicity (46) and attenuate drug hypersensitivity in mouse models (47). It is also notable that MAs are reported more frequently in patients with depression (48,49), anxiety (49), dissociative symptoms (50), and increased life stressors (51). Given that symptom overreporting has been demonstrated to be more frequent among patients with psychiatric diagnoses (52, 53), it is possible that patients with MAs are more likely to report symptoms at time of follow-up, accounting for the increase in late toxicity observed. However, these alternative biologic mechanisms and/or symptom overreporting would not explain the improvement in biochemical control seen in patients with MAs and therefore seem unlikely to be the sole cause of the differences in the long-term outcomes reported herein. Moreover, the large difference observed in severe toxicity requiring invasive procedures for correction further supports that the observed differences in late toxicity are not solely the result of increased somatization and symptom overreporting in patients with MAs.

This study is the first to our knowledge to associate MAs with RT treatment efficacy or toxicity. Several important caveats should be considered. Beyond the inherent limitations of any single-institution, retrospective study it is notable that the proportion of men with MAs in our cohort was relatively high, with 26.4% of men having at least 1 documented MA. The true incidence of MAs is not well defined in the general population, much less in men with prostate cancer; however, 3%-5% of hospitalized patients have been reported to have at least 1 MA (54). The higher proportion of MAs in our cohort is likely, at least partially, a function of the time period in which they were treated (1989-2006). Rates of MAs have declined in recent decades, as demonstrated by decreasing rates of skin prick test-confirmed penicillin allergies (55); however, it is notable that there was no statistically significant variation in rate of MA by year of treatment within our examined cohort. Moreover, given that men with prostate cancer are, on average, older than other members of the population, they may have had had increased medication exposure and more opportunity to identify MAs. It is also possible that MAs were more common than expected in our cohort as a result of patient reporting, which has been shown to result in higher rates of recorded MAs compared with when MAs are verified by physicians (56). It is also notable that the rate of late treatment–related toxicity in this cohort was higher compared with modern series. Since that time, advances in RT planning and delivery have decreased the risk of treatment-related toxicity (57,58), and any observed influence on toxicity may now be smaller with contemporary treatment. Finally, our study reported only physician-reported toxicity, which is known to potentially underestimate patient-reported outcomes (59). Although the use of physician-reported toxicity can potentially be discordant with patient experiences, our physician-reported outcomes help further support the conclusion that the observed differences in toxicity are not solely the result of increased somatization and symptom overreporting in patients with MAs.

In summary, our findings support the hypothesis that long-term outcomes following treatment with RT for prostate cancer are different in men with MAs and have the potential to influence the management of patients with localized prostate cancer, as well as other conditions treated with radiotherapy, pending further investigation. Given that the majority of patients with nonmetastatic prostate cancer are candidates for treatment with either primary RT or surgery and have excellent long-term disease-specific survival, potential toxicities and functional outcomes are important considerations when deciding between these approaches. If these results are validated, men with MAs who receive RT may be more strongly considered for treatment modifications such as the placement of a hydrogel rectal spacer and adoption of more stringent rectal sparing constraints, both of which have been demonstrated to reduce rates of long-term GU and GI morbidity (57,58,60). Furthermore, given the possible role of the adaptive T-cell response and downstream mediators such as TGF-β in late RT–related toxicity among patients with MAs, therapeutic approaches targeting these pathways warrant further study. For example, pentoxifylline, which downregulates TGF-β1, has attracted interest as a possible therapy for patients with scleroderma and post-RT fibrosis (61,62) and may be considered for further investigation in patients with MAs and late treatment–related morbidity.

Among men treated with primary RT for prostate cancer, men with MAs appeared to have a higher risk for late treatment–related toxicity and higher probability of disease control. Differences in long-term outcomes following RT between men with MAs and those without MAs may be explained by common underlying mechanisms between MA and RT effects in both tumor and normal tissues. Although these findings remain hypothesis-generating and require further validation, history of MA may be a relevant consideration in the counseling and treatment of men with localized prostate cancer.

## Funding

This study was funded internally with no external sources.

## Notes

**Role of the funder:** Not applicable.

**Disclosures:** The authors have no relevant conflicts of interest to disclose.

**Author contributions:** Conceptualization (WTT, SLL); Methodology (WTT, SLL); Formal analysis (WTT, SG, SLL); Investigation (WTT, SG, SLL); Resources (SLL); Data Curation (WTT, SG, SLL); Writing - Original Draft (WTT, SLL); Writing - Review & Editing (WTT, SG, MTS, SLL); Visualization (WTT); Supervision (SLL).

**Prior presentations:** These data have been accepted for presentation at the American Society for Radiation Oncology (ASTRO) Annual Meeting in October 2020; however, this manuscript has not been published and is not under consideration for publication elsewhere.

## Data availability statement

The data presented in this manuscript are not routinely available to other researchers (institutional database of human subjects and concern for data privacy).

## Supplementary Material

pkaa081_Supplementary_Data
