# Supplementary material for: Prostate Cancer Radiotherapy: Increased Biochemical Control and Late Toxicity in Men With Medication Allergies
Source: JNCI Cancer Spectr. 2020 Sep 11;4(6):pkaa081. doi: 10.1093/jncics/pkaa081 (PMC7771007; doi:10.1093/jncics/pkaa081)
Supplement: pkaa081_Supplementary_Data [file pkaa081_supplementary_data.pdf]

Supplementary Table 1. Univariate and multivariate analysis for 10-year freedom from distant metastasis (n = 587).

	UVA		MVA			
			Model 1		Model 2	
	10-year FFDM[f]	*P*-value	HR (95% CI)	*P*-Value	HR (95% CI)	*P*-Value
Pre-treatment PSA[a]						
< 10 ng/mL vs. 10 to < 20 ng/mL	94% vs. 93%	.746	-	-	1.15 (0.47-3.25)	.769
< 10 ng/mL vs. ≥ 20 ng/mL	94% vs. 81%	.002	-	-	0.78 (0.34-1.85)	.576
10 to < 20 ng/mL vs. ≥ 20 ng/mL	93% vs. 81%	.009	-	-	0.68 (0.22-1.89)	.469
Clinical T-stage						
T1a-T1c vs. T2a-T2c	94% vs. 80%	.009	-	-	0.41 (0.18-0.98)	.045
T1a-T1c vs. T3a-T3b	94% vs. 69%	<.001	-	-	0.23 (0.09-0.66)	.007
T2a-T2c vs. T3a-T3b	80% vs. 69%	.130	-	-	0.57 (0.20-1.67)	.290
Clinically Node-Positive						
No vs. Yes	91% vs. 50%	.372	-	-	-	-
ISUP[b] Grade Group						
1 vs. 2	96% vs. 84%	.003	-	-	0.31 (0.14-0.70)	.007
1 vs. 3	96% vs. 89%	.094	-	-	0.37 (0.11-1.26)	.137
1 vs. 4	96% vs. 80%	.001	-	-	0.31 (0.10-0.95)	.050
1 vs. 5	96% vs. 51%	<.001	-	-	0.18 (0.05-0.63)	.017
2 vs. 3	84% vs. 89%	.663	-	-	1.18 (0.35-4.03)	.785
2 vs. 4	84% vs. 80%	.567	-	-	1.00 (0.34-2.99)	.991
2 vs. 5	84% vs. 51%	.092	-	-	0.56 (0.16-1.99)	.389
3 vs. 4	89% vs. 80%	.436	-	-	0.85 (0.23-3.20)	.809
3 vs. 5	89% vs. 51%	.057	-	-	0.48 (0.11-2.06)	.325
4 vs. 5	80% vs. 51%	.376	-	-	0.56 (0.14-2.19)	.414
NCCN[c] Risk Category						
Low vs. Intermediate	97% vs. 93%	.192	0.59 (0.22-1.46)	.255	-	-
Low vs. High	97% vs. 80%	<.001	0.20 (0.08-0.49)	<.001	-	-
Intermediate vs. High	93% vs. 80%	.001	0.35 (0.17-0.69)	.002	-	-
Treatment Modality						
EBRT[d] Alone vs. Brachy[e] Alone	90% vs. 96%	.428	-	-	-	-
EBRT[d] Alone vs. EBRT[d] + Brachy[e]	90% vs. 96%	.641	-	-	-	-
Brachy[e] Alone vs. EBRT[d] + Brachy[e]	96% vs. 96%	.847	-	-	-	-
Dose Escalation (EBRT Dose ≥ 74 Gy/Brachy[e])						
Yes vs. No	92% vs. 90%	.261	-	-	-	-
Androgen Deprivation Therapy						
Yes vs. No	87% vs. 94%	.046	1.05 (0.56-2.02)	.876	1.20 (0.53-2.78)	.656
Medication Allergy						
Yes vs. No	95% vs. 89%	.104	0.58 (0.25-1.18)	.141	0.65 (0.27-1.40)	.286

PSA[a] = prostate specific antigen, ISUP[b] = International Society of Urological Pathology, NCCN[c] = National Comprehensive Cancer Network, EBRT[d] = external beam radiation therapy, Brachy[e] = brachytherapy, FFDM[f] = freedom from distant metastasis

Supplementary Table 2. Univariate and multivariate analysis for 10-year prostate cancer-specific survival (n = 587).

	UVA		MVA			
			Model 1		Model 2	
	10-year PCSS[f]	*P*-value	HR (95% CI)	*P*-Value	HR (95% CI)	*P*-Value
Pre-treatment PSA[a]						
< 10 ng/mL vs. 10 to < 20 ng/mL	95% vs. 98%	.093	-	-	5.71 (1.11-104.60)	.035
< 10 ng/mL vs. ≥ 20 ng/mL	95% vs. 90%	.009	-	-	0.79 (0.30-2.20)	.641
10 to < 20 ng/mL vs. ≥ 20 ng/mL	98% vs. 90%	.002	-	-	0.14 (0.08-0.81)	.025
Clinical T-stage						
T1a-T1c vs. T2a-T2c	96% vs. 91%	.055	-	-	0.73 (0.25-2.43)	.590
T1a-T1c vs. T3a-T3b	96% vs. 86%	.001	-	-	0.36 (0.10-1.66)	.168
T2a-T2c vs. T3a-T3b	91% vs. 86%	.299	-	-	0.49 (0.11-2.51)	.361
Clinically Node-Positive						
No vs. Yes	95% vs. 100%	.624	-	-	-	-
ISUP[b] Grade Group						
1 vs. 2	96% vs. 94%	.923	-	-	1.01 (0.28-3.67)	.982
1 vs. 3	96% vs. 93%	.664	-	-	0.43 (0.09-2.09)	.335
1 vs. 4	96% vs. 94%	.524	-	-	0.69 (0.14-3.35)	.655
1 vs. 5	96% vs. 80%	<.001	-	-	0.15 (0.04-0.57)	.018
2 vs. 3	94% vs. 93%	.795	-	-	0.42 (0.07-2.68)	.378
2 vs. 4	94% vs. 94%	.520	-	-	0.68 (0.11-4.23)	.681
2 vs. 5	94% vs. 80%	.002	-	-	0.14 (0.03-0.74)	.029
3 vs. 4	93% vs. 94%	.826	-	-	1.60 (0.21-12.16)	.650
3 vs. 5	93% vs. 80%	.038	-	-	0.34 (0.05-2.22)	.254
4 vs. 5	94% vs. 80%	.074	-	-	0.21 (0.03-1.38)	.103
NCCN[c] Risk Category						
Low vs. Intermediate	95% vs. 98%	.201	2.46 (0.68-11.48)	.172	-	-
Low vs. High	95% vs. 90%	.002	0.33 (0.13-0.80)	.013	-	-
Intermediate vs. High	98% vs. 90%	<.001	0.13 (0.03-0.41)	<.001	-	-
Treatment Modality						
EBRT[d] Alone vs. Brachy[e] Alone	95% vs. 97%	.433	-	-	-	-
EBRT[d] Alone vs. EBRT[d] + Brachy[e]	95% vs. 96%	.745	-	-	-	-
Brachy[e] Alone vs. EBRT[d] + Brachy[e]	97% vs. 96%	.440	-	-	-	-
Dose Escalation (EBRT Dose ≥ 74 Gy/Brachy[e])						
Yes vs. No	96% vs. 94%	.091	0.67 (0.29-1.48)	.325	0.46 (0.18-1.14)	.093
Androgen Deprivation Therapy						
Yes vs. No	95% vs. 95%	.894	-	-	-	-
Medication Allergy						
Yes vs. No	95% vs. 95%	.244	0.61 (0.20-1.51)	.306	0.66 (0.21-1.75)	.426

PSA[a] = prostate specific antigen, ISUP[b] = International Society of Urological Pathology, NCCN[c] = National Comprehensive Cancer Network, EBRT[d] = external beam radiation therapy, Brachy[e] = brachytherapy, PCSS[f] = prostate cancer-specific survival
